# Supplementary material for: Optimization and control of actuator networks in variable geometry truss systems using genetic algorithms
Source: Nat Commun. 2025 Sep 30;16:8432. doi: 10.1038/s41467-025-63373-7 (PMC12484673; doi:10.1038/s41467-025-63373-7)
Supplement: Supplementary file 1 — Supplementary Information [file 41467_2025_63373_MOESM1_ESM.pdf]

Supplementary Information

Optimization and Control of Actuator Networks in Variable Geometry Truss Systems Using Genetic Algorithms

Jianzhe Gu[1,2], Ziwen Ye[2], Tucker Rae-Grant[2], Shuhong Wang[1,2], Ding Zhao[4], Josiah Hester[3], Victoria A. Webster-Wood[4], Lining Yao[1,2*]

[1]Morphing Matter Lab, Mechanical Engineering, University of California, Berkeley, California, USA
[2]Human-Computer Interaction Institute, Carnegie Mellon University, Pittsburgh, Pennsylvania, USA
[3]Interactive Computing and Computer Science, Georgia Institute of Technology, Atlanta, Georgia, USA
[4]Mechanical Engineering, Carnegie Mellon University, Pittsburgh, Pennsylvania, USA
[*]Correspondence to: liningy@berkeley.edu

This PDF file includes:

Supplementary Note 1: Simulator Details

Supplementary Note 2: Simulator Comparison

Supplementary Note 3: Representation Details

Supplementary Note 4: NSGA-II Explanation

Supplementary Note 5: Objective Functions

Supplementary Note 6: On-body Control Circuit

Supplementary Note 7: Algorithms

Supplementary Figure 1: Truss symmetry definition

Supplementary Figure 2: Metatruss Mechanism

Video S1: A quadruped metatruss performs walking, turning around, lowering body and tilting the top. (Left: perspective view, Right: side or top view.)

Video S2: Top: A lobster metatruss performs walking. Bottom: The same lobster metatruss

performs walking with energy efficiency. Right: Training result showing the relationship between the hypervolume of the multi-objective optimization for the lobster metatruss.

Video S3: A Helmet metatruss transforms into two target shapes from the same initial shape. (Left: perspective view, Right: side or top view.)

Video S4: A tentacle metatruss reaching three different target positions. (Left: perspective view, right: side view.)

Video S5: Top: tracked video of a fabricated pillbug metatruss walking forward. Bottom: The simulation of the pillbug metatruss walking forward.

Table S1: Tukey's HSD Pairwise Group Comparisons (95.0% Confidence Interval) for the hypervolume performance of quadruped robot with 5 C-network counts.

Supplementary Note 1: Simulator Details

In our simulation, we use a dynamic mass-spring model with high damping coefficient (Supplementary Figure 1). The simulator captures the gravity, friction, and axial forces of beams. As friction plays a crucial role in the metatruss performance, a dynamic simulator is chosen over a semi-static simulation. Due to asynchronized actuation, we keep the motion of the system as static as possible to avoid unpredictable asynchronized dynamics from actuation. Thus, a high damping coefficient is used to capture the semi-static nature of the motion. Considering the existence of friction and semi-static motion, we use a mass-spring model with an explicit integrator and high damping coefficient. The simulation consists of K steps within a transformation period. During each step, incremental adjustments are made to the rest lengths of beams, transitioning them from their initial length l_{ij_0} to their target lengths l_{ij_g}. Specifically, the rest length $l_{ij}{}_k^*$ between joints i, j at the kth step of a transformation period is calculated as

$$l_{ij}{}_k^* = (1 - \frac{k}{K}) l_{ij_0} + \frac{k}{K} l_{ij_g} \tag{1}$$

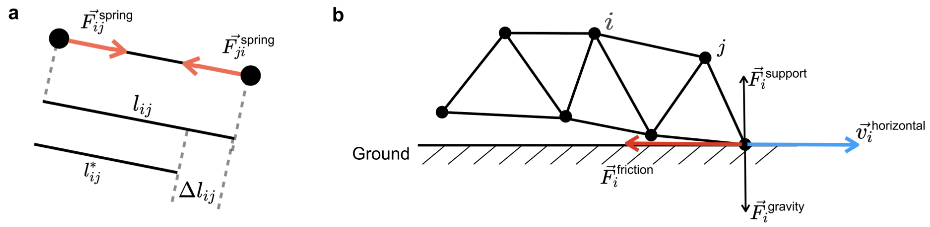

Supplementary Figure 1: Illustration of the simulator. **a**, Spring force vectors on beam joints, applicable when beam length l_{ij} exceeds rest length l_{ij}^*. **b**, Force vectors illustration when the metatruss contacts the ground with horizontal velocity. In addition to the spring forces, each joint receives a gravity force. On ground contact, a joint encounters a vertical upward support force and a friction force in the opposite direction of the horizontal velocity component.

Each step of simulation is further divided into multiple sub-steps, where force, velocity, and position updates are performed.

Let $\vec{p}_i$ be the position vector of point i. Let $\vec{v}_i$ be the velocity vector of point i. Let k be the spring constant, and Δl_{ij} be the difference between the actual length l_{ij} and the rest length l_{ij}^* of the spring between points i and j. Let $\vec{g}$ be the acceleration due to gravity. Let γ be the damping coefficient. Let μ be the friction coefficient.

Spring Force. Spring forces aim to maintain a certain rest length between each connected pair of points:

$$\vec{F}_{ij}^{\text{spring}} = -k \Delta l_{ij} \hat{r}_{ij} \tag{2}$$

where k is the spring constant, Δl_{ij} is the difference between the actual length l_{ij} and the rest length l_{ij}^*, and $\hat{r}_{ij}$ is the unit vector from i to j.

Gravitational Force. Gravitational forces act uniformly downward on all points, defined as

$$\vec{F}_i^{\text{gravity}} = m_i \vec{g} \tag{3}$$

where $\vec{g}$ represents the acceleration of gravity.

Support Force. When a joint contacts the ground, support forces counteract gravity:

$$\vec{F}_i^{\text{support}} = -\min(0, \vec{F}_i \cdot \hat{z})\hat{z} \tag{4}$$

where $\hat{z}$ is the unit vector perpendicular to the ground pointing upwards, and $\vec{F}_i$ is the total force on the ith joint.

Frictional Force (when in contact with the ground). Frictional forces are dependent on the horizontal velocity of the points in contact with the ground

$$\vec{F}_i^{\text{friction}} = -\mu \vec{v}_{i,\text{horizontal}} \tag{5}$$

where μ is the friction coefficient.

Total Force The total force on a point i is the sum of all individual forces:

$$\vec{F}_i = \vec{F}_i^{\text{spring}} + \vec{F}_i^{\text{gravity}} + \vec{F}_i^{\text{support}} + \vec{F}_i^{\text{friction}} \tag{6}$$

Explicit Integration for Velocity and Position

1. Update velocity:

$$\vec{v}_i(t + \Delta t) = \vec{v}_i(t) + \frac{\vec{F}_i}{m_i}\Delta t - \gamma \vec{v}_i(t)\Delta t \tag{7}$$

   where γ is the damping coefficient.

2. Update position:

$$\vec{p}_i(t + \Delta t) = \vec{p}_i(t) + \vec{v}_i(t + \Delta t)\Delta t \tag{8}$$

   where $\vec{p}_i$ is the position vector of the ith joint, $\vec{v}_i$ is the velocity vector of the ith joint.

Supplementary Note 2: Simulator Comparison

To validate our simulator's accuracy and efficiency, we implemented a comparative model using MuJoCo, a widely used physics engine renowned for its precision in robotics simulation. In the MuJoCo implementation, each linear actuator is modeled using a prismatic slide joint that incorporates a sliding constraint and integrates spring forces directly via MuJoCo's built-in force elements with specified stiffness and damping parameters. The connections between actuators and vertices are implemented using ball joints that provide three rotational degrees of freedom for the connection between the joints and the linear actuators. All simulation parameters, including step size, gravitational constants, mass distributions, and friction coefficients, were maintained consistently across both simulators to ensure a rigorous comparison.

We compared the simulators across multiple test cases: four quadruped motions, two pillbug motions, and three tentacle positioning tasks. For each test case, we calculated the RMSE between corresponding joint positions along their trajectories, tracking the deviation between simulators at each timestep. We then normalized these RMSE values by the total displacement of each joint's motion and averaged across all joints. This normalized RMSE showed an average difference of 3.60% between simulators. For a joint trajectory with 100mm total displacement, this represents a 3.6mm average deviation between simulators, demonstrating strong agreement across diverse locomotion patterns.

Performance analysis showed significant speed advantages in our custom simulator. For a standardized test of 10,000 timesteps, our simulator completed the computation in 0.12 seconds, compared to MuJoCo's 40.80 seconds. This show an over 340 times improvement in computational efficiency. This substantial speed increase can be attributed to two main factors: our simplified physics model that focuses on essential dynamics relevant to the metatruss system, and our optimized C++ implementation designed specifically for truss-like structures.

Supplementary Note 3: Representation Details

We define a metatruss structure as a tuple (V, E), where:

$V = \{v_i\}_{i=0}^{N_V-1}$ is the set of joints (named after vertices), with each joint $v_i \in \mathbb{R}^3$ indicating a position in three-dimensional space.

$E = \{e_i\}_{i=0}^{N_E-1}$ is the set of beams (named after edges), with each beam $e_i = \{j_{i0}, j_{i1}\}$ representing an unordered pair of distinct vertices with indices $j_{i0}, j_{i1} \in \{0, 1, \ldots, N_V - 1\}$.

A metatruss design includes a tuple (C, L), where:

$C = \{c_i\}_{i=0}^{N_E-1}$ is the set of C-network indices, with each C-network index c_i corresponding to the beam e_i and $c_i \in \{0, 1, \ldots, N_C - 1\}$, where N_C is the total number of C-networks.

$\Lambda = \{\lambda_i\}_{i=0}^{N_E-1}$ denotes the contraction levels for each beam. Each λ_i corresponds to the beam e_i and takes a value from the set $\{0, 1, \ldots, N_\Lambda - 1\}$, where N_Λ indicates the total number of contraction levels.

During actuation, each beam can contract by a predefined contraction ratio r, which equals the product of its contraction level λ and contraction increment Δ. The contraction increment is a constant value within $(0, \frac{1.0}{N_\Lambda-1})$. In our work, we set $\Delta = 0.12$ and $N_\Lambda = 4$. Hence, the possible contraction ratios are in the set $\{0\%, 12\%, 24\%, 36\%\}$. This means that, depending on its contraction level, each beam can contract by one of these ratios. In practice, the values of Δ and λ are often determined by specific hardware constraints.

We take the C-network indices C, contraction levels Λ, and actuation sequences $A_0, A_1, ..A_{N_A}$, flatten them, and concatenate them into a 1D integer vector as a representation of a truss configuration D. This encoding represents all the information of a metatruss design as an integer vector that is suitable for the genetic algorithm to optimize.

We define $A_j = \{a_i\}_{i=0}^{N_{Aj}-1}$ as the actuation sequence for the metatruss, where N_{Aj} is the number of actions in the jth action sequence. During simulation, the metatruss will take in actions in sequence and actuate the truss. Each action $a_i \in \{0, 1\}^{N_C}$ controls the on or off state of each C-network. During the actuation of each action, beams in each C-network expand or contract based on the action. τ is the time duration of each action. The total actuation time T equals to $\tau \cdot N_{Aj}$ for the jth action sequence A_j.

A metatruss goes through a set of action sequences $\{A_0, A_1, \ldots, A_{N_A-1}$, where each action sequence A_i is expected to achieve one task S_i. A task S_i consists of a set of subtasks. For example, $S_i = \{s_l, s_t\}$ represents the metatruss performs locomotion and turning under the action sequence of the ith task.

Supplementary Note 4: NSGA-II Explanation

NSGA-II introduces metrics to find the best-performing group on multi-objectives. It uses non-dominant sorting and crowding distance sorting. First, it calculates the rank (R) of a design based on whether it is dominated by other designs. The definition of dominance is as follows: Given a design 1 with multi-objective fitness $S_1 = s_{1,0}, s_{1,1}, ..., s_{1,n}$, and a design 2 with fitness $S_2 = s_{2,0}, s_{2,1}, ..., s_{2,n}$, if

$$s_{2,i} \geq s_{1,i} \forall i \in 0, 1, \ldots, n \text{ and } \exists j : s_{2,j} > s_{1,j}$$

we say that design 2 dominates design 1. Designs that are not dominated by any other design have rank 0. To determine rank 1, we consider only the designs not in rank 0, and find those that are not dominated within this subset. We continue this process to find subsequent ranks (Supplementary Figure 2a). The $R = 0$ designs form the Pareto set, and their fitness values form the Pareto front.

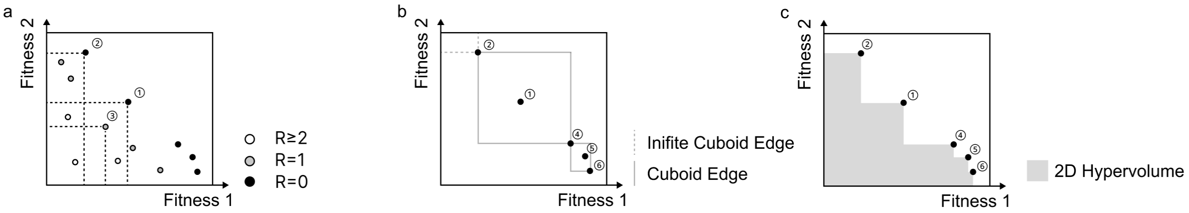

Supplementary Figure 2: NSGA-II explanation for a two-fitness optimization problem. a, Non-dominated sorting: Designs 1 and 2 have $R = 0$ as they are not dominated by any other design. Design 3 is dominated by Design 1 but not by any other non-$R = 0$ design, so it has $R = 1$. **b**, Crowding distance calculation: For each design, the cuboid formed by its nearest neighbors in the fitness space is considered. The crowding distance is the sum of the normalized side lengths of this cuboid. For example, $CD_1 = \frac{d_{1,2}}{f_2^{max} - f_2^{min}} + \frac{d_{1,1}}{f_1^{max} - f_1^{min}}$, where $d_{1,2}$ and $d_{1,1}$ are the distances to the nearest neighbors in fitness dimensions 2 and 1, respectively. **c**, Hypervolume: The hypervolume (area in 2D) covered by the Pareto front, which serves as a measure of the quality and diversity of non-dominated designs.

The crowding distance (CD) is calculated to further sort designs within the same rank and to encourage diversity. CD measures how crowded it is around a design in the fitness space. It is calculated as follows: For a design i in a particular front:

For each fitness dimension m: a. Sort the designs in the front by fitness m. b. Assign infinite distance to boundary designs. c. For all other designs, assign a distance equal to the absolute normalized difference in the fitness values of two adjacent designs. The overall CD for design i is the sum of individual distance values for each fitness dimension:

$$CD_i = \sum_{m=1}^{M} \frac{f_m(i+1) - f_m(i-1)}{f_m^{max} - f_m^{min}}$$

where M is the number of fitness dimensions, $f_m(i)$ is the m-th fitness value of the i-th design, and f_m^{max} and f_m^{min} are the maximum and minimum values of the m-th fitness dimension. A larger CD implies that the design is more unique and potentially has distinct features, adding to the diversity of

the generation. During the selection process, designs are first sorted by rank and then by crowding distance (Supplementary Figure 2b).

To evaluate the overall performance of a generation in multi-objective optimization, we use the hypervolume metric (Supplementary Figure 2c). The hypervolume is the n-dimensional space covered by the Pareto front with respect to a reference point. In the case of two fitness dimensions, it represents the area dominated by the Pareto front. A larger hypervolume indicates better overall performance and diversity of the non-dominated designs. The hypervolume provides a single scalar value that captures both the quality of the solutions and their spread across the Pareto front, making it a useful metric for comparing different generations or optimization algorithms.

Supplementary Note 5: Objective Functions

Walking. The walking subtask is defined as maximizing the directional displacement of the robot. The centroid position p_c is the position of the center of mass of all joints. At time t, the centroid position is computed as $p_c(t) = \frac{1}{N_V} \sum_{i=0}^{N_V-1} v_i(t)$. During a given period of time expressed by $N_T \cdot I$, the displacement of the centroid along x axis is defined as the walking distance $\tilde{d_w}$. The robot is expected to achieve a walking distance as large as possible. u_x is the unit vector along the x axis.

$$\tilde{d_w}(t) = (p_c(t) - p_c(0)) \cdot u_x \tag{9}$$

Reorientation The reorientation subtask is to maximize the orientation alignment $\tilde{a_o}$ between the original orientation of a subset of the metatruss, represented as a unit vector u_o, with the target orientation, represented as a unit vector u_g. In this case, the metatruss starts with an initial orientation represented as a unit vector $u_o(0)$, and is expected to align with a target unit vector u_g. A subset of vertices V_o in the metatruss are assigned to determine the orientation. For the turning subtask of the quadruped robot shown in Figure 1d, five vertices in two incident tetrahedrons in the middle part of the robot are assigned as V_o to represent the body. $u_o(0)$ is assigned as the unit vector along the positive direction of the x-axis, while u_g is the unit vector along the positive y-axis. We define the orientation alignment fitness value as

$$\tilde{a_o} = u_o \cdot u_g \tag{10}$$

As the metatruss deforms, the vertices are subject to relative movement, we determine the metatruss's orientation by calculating a transformation matrix. This matrix transforms the initial vertex positions, demoted as $V_o(0)$, to approximate their final positions $V_o(T)$ at time T. We then extract the orientation information from this transformation. To achieve this, we employ the BFGS optimizer [70] to optimize the 3D transformation matrix $\mathcal{F}$. This optimization minimizes the average distance between the current positions $V_o(t)$ and the transformed initial positions $V_o(0) \cdot \mathcal{F}$. Consequently, we derived the current orientation $u_o(T)$ by rotating the initial orientation $u_o(0)$ with $\mathcal{F}$.

Energy Efficiency during Walking. This function is defined as minimizing the average energy consumption during walking for a unit distance. Here the total energy consumption is calculated by summing up the work done by all the beams. The work is calculated by summing up the product of axial force f of each beam and its length change Δ_l at every time step. The energy efficiency fitness value $\tilde{\phi}$ is defined as the negative of the quotient obtained by dividing the total energy consumption by the total directional displacement as described in equation (9).

$$\tilde{\phi} = -\frac{\sum_{i=0}^{N_E-1} \sum_{t=0}^{N_T \cdot I-1} \Delta_{l_i}(t) \cdot f_i(t)}{\tilde{d_w}(t)} \tag{11}$$

Here, i indexes each beam, and t represents the time step.

Shape Approximation. The shape approximation subtask guides the metatruss to approximate a target shape, prioritizing selected key joints to reach target locations while considering the spatial relationships among all joints. As the morphing helmet example (Fig. 7a) shows, in two objective functions, three joints are designated as key joints including one each at the front, left, and right. Their are used to guide the deformation optimization.

The subtask is formulated as minimizing the sum of the mean squared distances between the joints and their respective target positions, with a weight applied to all joints. First, a set of key joints $I_k = \{i_0, i_1, \ldots, i_{N_X-1}\}$ is selected, and their target positions $P_t = \{p_{t0}, p_{t1}, \ldots, p_{tN_X-1}\}$ are defined. For non-key joints, their target positions are set to be their initial positions and a weight is applied based on proximity to the closest key joint along the beams. Figure 7b demonstrates the weighting scheme, where the weight for each joint diminishes as it moves closer to the key joints, and key joints themselves carry zero weight.

Specifically, the weight for each non-key joint is determined by the distance d_i to its closest key joint along the beams, with the accumulated beam lengths defining the distance. The distance is then converted to weight using a hyperbolic tangent transformation: $w_i = \frac{1}{\pi} \tan^{-1}((d_i - \alpha) \cdot \beta) + \frac{1}{2}$, where d_i is the length of the shortest path to the closest key joint, and α and β are constants. In the context of the helmet (Fig. 7a-c), the $\alpha = 2.3, \beta = 2.0$.

The shape approximation fitness value X is defined as:

$$X(t) = -\left(\frac{1}{N_X} \sum_{i \in I_k} \|v_i(t) - p_{t_i}\|_2 + \xi \cdot \sum_{i=0}^{N_V-1} w_i \|v_i(t) - v_i(0)\|_2\right) \tag{12}$$

Here, ξ indicates the importance of keeping non-key joints to their original positions. In the case of helmet, ξ is set to 1.0 to keep the overall shape, while in the case of tentacle, ξ is set to 0.0 to maximize the range of accuracy of the key joint.

Here, the metatruss maintains its overall shape while adjusting key joints to align with the target positions. This leads to a controlled deformation that respects the spatial relationships between joints.

Supplementary Note 6: On-body Control Circuit Using Mechanical Logic Gates

Our optimization generates sequential open-loop control signals that could potentially be implemented through mechanical structures, eliminating the need for multiple electronic control inputs and requiring only a constant power supply. Taking our pillbug's forward walking task as an example, the optimized open-loop control sequence consists of a 4-bit binary signal over 4 time steps (Supplementary Figure 3a). Each time step's control signal can be represented as a 2-bit input generating a 4-bit output, making it implementable through a 2-to-4 multiplexer (Supplementary Figure 3b). We used logic optimization tools to analyze the truth table and derived a simplified circuit for the Pillbug's actuation sequence. The resulting circuit requires 14 logic gates, including one clock unit (Supplementary Figure 3c).

For physical implementation, we identified a mechanical circuit design from previous work [62, 63] that uses pneumatic power and cylindrical units with bi-stable membranes. In this design, the air pressure differential between two chambers affects the membrane's direction (Supplementary Figure 3d,e), which in turn blocks one of the air tubings. By connecting the six ports to different pressure sources, each unit can be preconfigured to function as AND, OR, or NOT gates (Supplementary Figure 3f), theoretically enabling the implementation of any logic circuit. Additionally, when connected to an external tank and a constant air pressure source, these units can function as oscillators, serving as the clock component in the logic circuit (Supplementary Figure 3g).

While current mechanical logic units measure approximately 4cm, they have potential for miniaturization. Similarly, our metatruss design can be scaled up through modified fabrication processes and corresponding parameter adjustments in the simulator, without requiring changes to the optimizer. Assuming mechanical logic units could be reduced to 1cm^3 and metatruss beams could be expanded to 20cm length, we created renderings of a circuit board containing all required units and visualized a pillbug robot carrying this control system (Supplementary Figure 3h). In this configuration, the robot would require only a single constant air power source, enabling operation either through a single tethered air tube or potentially untethered if equipped with an onboard compressed air tank.

This implementation strategy demonstrates how our metatruss system could evolve toward more self-contained, mechanically controlled robots with simplified power and control requirements.

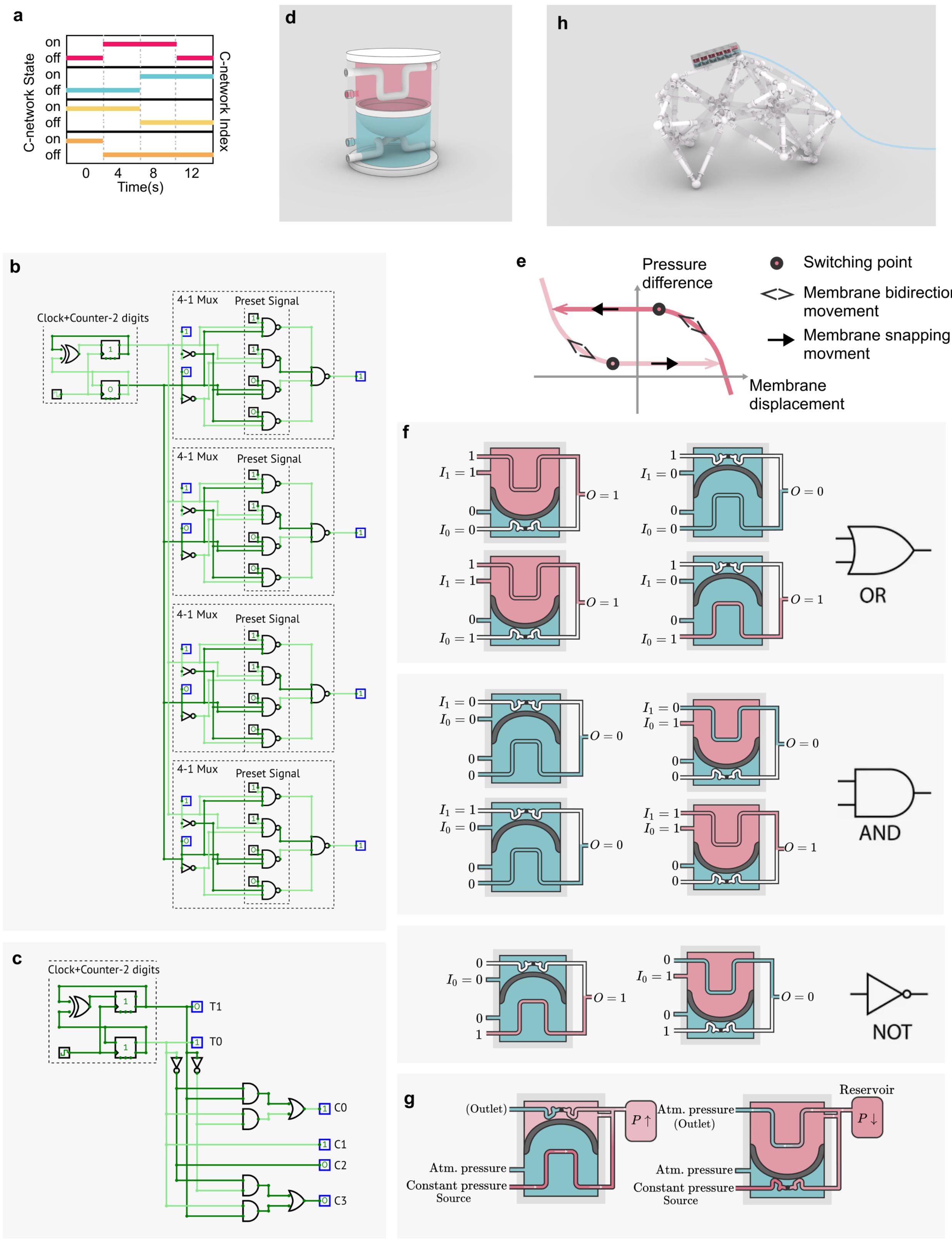

Supplementary Figure 3: On-body control circuit using mechanical logic gates. **a**, Open-loop control signal optimized for Pillbug metatruss towards walking forward. **b**, 4-to-8 multiplexer circuit for the open-loop signal. **c**, Simplified circuit for the open-loop signal. **d**, The basic mechanical circuit unit. **e**, The illustration of the bi-stable mechanism of the mechanical circuit unit. **f**, The illustration of the ports connection for AND, OR, and NOT gate. **g**, The illustration of the clock unit. **h**, A rendering of the pillbug with on-body mechanical open-loop control circuit.

Supplementary Note 7: Algorithms

Here we describe four algorithms critical to our computational pipeline, including the algorithm for initializing the indices of the C network, the algorithm for mutation, the algorithm for the constrained crossover operator and the algorithm for elitism-only training iterations.

Box 1 — Random Initialization of C-network Indices

1. Initialize sets: Define $I_C = \{0, 1, \ldots, N_C - 1\}$ as the set of all C-network indices. Let E_u denote unassigned beams, E_i denote beams of the ith C-network, E_s denote self-symmetric beams, and E_r denote inter-symmetric beams.

2. Assign symmetric pairs: For each C-network c_i where $i \in I_C$:

- If there exists $c_{i'}$ such that $c_i \leftrightarrow c_{i'}$, randomly select $e, e' \in E_r$ where $e \leftrightarrow e'$. Move e from E_u to E_i and e' from E_u to $E_{i'}$.
- Else if $\odot c_i$, randomly select $e \in E_s$ and move e from E_u to E_i.

3. Assign remaining beams: Repeat the following until E_u is empty:

- Randomly select $e \in E_u$ adjacent to any assigned beam.
- If $\odot e$, randomly select an adjacent C-network i such that $\odot i$, then move e from E_u to E_i.
- If there exists e' such that $e' \leftrightarrow e$:
  - Randomly select an adjacent C-network index i.
  - If there exists i' such that $i' \leftrightarrow i$, move e from E_u to E_i and e' from E_u to $E_{i'}$.
  - Otherwise, move both e and e' from E_u to E_i.

Box 2 — Mutation of C-network Indices

1. Initialize parameters: Define p_m as the probability of executing mutation, I_s as self-symmetric C-network indices, and I_r as inter-symmetric C-network indices. Generate a random number $r \in [0, 1]$.

2. Mutation process: Repeat the following steps until $r \geq p_m$:

2a. Execute mutation: If $r < p_m$:

- Randomly select beam $e \in E$ with current C-network index i.
- Identify all adjacent C-network indices I_a.
- Apply mutation based on beam symmetry:
  - *Self-symmetric case:* If $\odot e$, randomly select a C-network with index $i_m \in I_s \cap I_a$ and move e from E_i to E_{i_m}.
  - *Inter-symmetric case:* If there exists e' such that $e' \leftrightarrow e$:
    - ∗ Randomly select C-network $i_m \in I_a$.
    - ∗ If there exists i'_m such that $i'_m \leftrightarrow i$, move e to E_{i_m} and e' to $E_{i'_m}$.
    - ∗ If $\odot i_m$, move both e and e' to E_{i_m}.

2b. Validate connectivity: Check if E_i or E_{i_m} becomes disconnected after the mutation. If disconnection occurs, revert the C-network assignment to maintain network integrity.

2c. Continue iteration: Generate a new random number $r \in [0, 1]$ and proceed to the next iteration.

Box 3 — Constrained Crossover of C-network Indices

1. Initialize parameters: Define p_c as the probability of executing crossover and select two survived designs D^0 and D^1 for crossover. Let E^0 and E^1 denote the sets of beams in D^0 and D^1 respectively, where e_i^j represents the ith beam of the jth selected design. Generate a random number $r \in [0, 1]$.

2. Crossover process: Repeat the following steps until $r \geq p_c$:

2a. Primary beam swap:

- Randomly select beam $e_i^0 \in E^0$ with C-network index j_0.
- Select corresponding beam $e_i^1 \in E^1$ with C-network index j_1.
- Swap the C-network indices by moving e_i^0 from $E_{j_0}^0$ to $E_{j_1}^0$ and moving e_i^1 from $E_{j_1}^1$ to $E_{j_0}^1$.

2b. Handle inter-symmetric beams: If there exist beams $e_{i'}^0$ and $e_{i'}^1$ such that $e_{i'}^0 \leftrightarrow e_i^0$ and $e_{i'}^1 \leftrightarrow e_i^1$, swap the C-network indices of these inter-symmetric beams by moving $e_{i'}^0$ from $E_{j'_0}^0$ to $E_{j'_1}^0$ and moving $e_{i'}^1$ from $E_{j'_1}^1$ to $E_{j'_0}^1$.

2c. Validate connectivity: Check if any C-network E_k^0 or E_k^1 (where $k \in I_C$) becomes disconnected after the swap operation. If disconnection occurs, revert the swap of C-network indices to maintain structural integrity.

2d. Continue iteration: Generate a new random number $r \in [0, 1]$ and proceed to the next iteration.

Box 4 — Single Training Iterations with Elitism

1. Initialize parameters: Define N_p as the maximum number of designs in a pool, N_s as the number of designs that survive, N_m as the number of designs generated by mutation, N_c as the number of designs generated by crossover, and N_i as the number of iterations in each generation. Let P_e denote the elite pool and P_a denote the active pool.

2. Pool initialization:

- If the cardinality of P_e equals N_p, move N_p designs from P_e to P_a.
- Otherwise, initialize N_p new designs in P_a.

3. Training iterations: For each iteration $i = 0, 1, \ldots, N_i - 1$:

3a. Fitness evaluation and selection:

- Evaluate the fitness of all designs in P_a as $f_i, \forall i \in P_a$.
- Apply NSGA-II sorting to P_a, resulting in sorted designs $P_a = \{D_0, D_1, \ldots, D_{N_p-1}\}$ with corresponding fitness $\{\hat{f_0}, \hat{f_1}, \ldots, \hat{f_{N_p-1}}\}$.
- Retain only the top N_s designs: $P_a \leftarrow \{D_0, D_1, \ldots, D_{N_s-1}\}$.

3b. Population generation:

- *Mutation:* For $j = 0, 1, \ldots, N_m - 1$, select a design $D_m \in P_a$ uniformly at random and add its mutation $\bar{D_m}$ to P_a.
- *Crossover:* For $j = 0, 1, \ldots, N_c - 1$, select two distinct designs $D_{c0}, D_{c1} \in P_a$ uniformly at random and add the crossover result $\bar{D_c}$ to P_a.
- *New initialization:* For $j = 0, 1, \ldots, N_i - (N_m + N_c) - 1$, initialize a new design $\bar{D_i}$ and insert into P_a.

4. Elite pool update: Move the top N_s designs from P_a to P_e and clear P_a.

Supplementary Figure 4: Truss symmetry definition

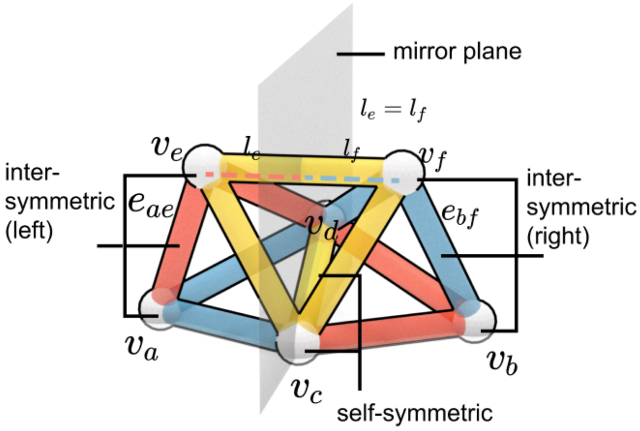

Supplementary Figure 4: Truss symmetry definition. v_e and v_f are inter-symmetric joints because they are mirrored against the mirror plane. Similarly, v_b and v_a are inter-symmetric, while v_c and v_d are self-symmetric as they are at the mirror plane. With both joints inter-symmetric to another beam's joints, e_{ae} is inter-symmetric to e_{bf}. Blue C-network is inter-symmetric to red C-network, while yellow C-network is self-symmetric.

Supplementary Figure 5:Metatruss Mechanism

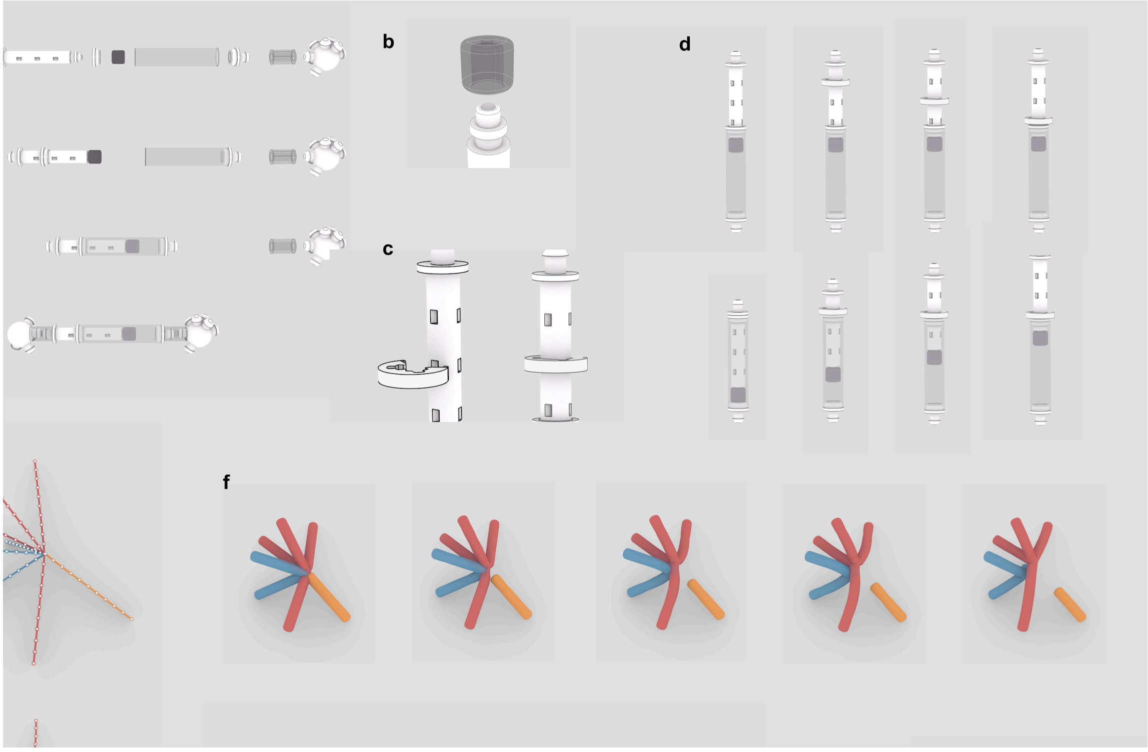

Supplementary Figure 5: Metatruss Mechanism

Supplementary Videos

Video S1: A quadruped metatruss performs walking, turning around, lowering body and tilting the top. (Left: perspective view. Right: side or top view.)

Video S2: Top: A lobster metatruss performs walking. Bottom: The same lobster metatruss performs walking with energy efficiency. Right: Training result showing the relationship between the hypervolume of the multi-objective optimization for the lobster metatruss.

Video S3: A Helmet metatruss transforms into two target shapes from the same initial shape. (Left:

perspective view, Right: side or top view.)

Video S4: A tentacle metatruss reaching three different target positions. (Left: perspective view, right: side view.)

Video S5: Top: tracked video of a fabricated pillbug metatruss walking forward. Bottom: The simulation of the pillbug metatruss walking forward.

Supplementary Table

Table S1: Tukey's HSD Pairwise Group Comparisons (95.0% Confidence Interval) for the hypervolume performance of quadruped robot with 5 C-network counts, with group 0 for 2 C-networks, group 1 for 8 C-networks, 2 for 16 C-networks, 3 for 32 C-networks, 4 for 64 C-networks

Comparison	Statistic	p-value	Lower CI	Upper CI
(0 - 1)	-12.799	0.003	-22.023	-3.574
(0 - 2)	-15.764	0.000	-24.989	-6.540
(0 - 3)	-18.710	0.000	-27.935	-9.486
(0 - 4)	-19.695	0.000	-28.919	-10.470
(1 - 0)	12.799	0.003	3.574	22.023
(1 - 2)	-2.965	0.877	-12.190	6.259
(1 - 3)	-5.911	0.352	-15.136	3.313
(1 - 4)	-6.896	0.214	-16.120	2.329
(2 - 0)	15.764	0.000	6.540	24.989
(2 - 1)	2.965	0.877	-6.259	12.190
(2 - 3)	-2.946	0.879	-12.170	6.279
(2 - 4)	-3.930	0.722	-13.155	5.294
(3 - 0)	18.710	0.000	9.486	27.935
(3 - 1)	5.911	0.352	-3.313	15.136
(3 - 2)	2.946	0.879	-6.279	12.170
(3 - 4)	-0.985	0.998	-10.209	8.240
(4 - 0)	19.695	0.000	10.470	28.919
(4 - 1)	6.896	0.214	-2.329	16.120
(4 - 2)	3.930	0.722	-5.294	13.155
(4 - 3)	0.985	0.998	-8.240	10.209
