# Supplementary material for: Optimization and control of actuator networks in variable geometry truss systems using genetic algorithms
Source: Nat Commun. 2025 Sep 30;16:8432. doi: 10.1038/s41467-025-63373-7 (PMC12484673; doi:10.1038/s41467-025-63373-7)
Supplement: Supplementary file 2 — Description of Additional Supplementary Files [file 41467_2025_63373_MOESM2_ESM.pdf]

Description of Additional Supplementary Files

Supplementary Video S1: A quadruped metatruss performs walking, turning around, lowering body and tilting the top. (Left: perspective view, right: side or top view.)

Supplementary Video S2: Top: A lobster metatruss performs walking. Bottom: The same lobster metatruss performs walking with energy efficiency. Right: Training result showing the relationship between the hypervolume of the multi-objective optimization for the lobster metatruss

Supplementary Video S3: A Helmet metatruss transforms into two target shapes from the same initial shape. (Left: perspective view, right: side or top view.)

Supplementary Video S4: A tentacle metatruss reaching three different target positions. (Left: perspective view, right: side view.)

Supplementary Video S5: Top: tracked video of a fabricated pillbug metatruss walking forward. Bottom: The simulation of the pillbug metatruss walking forward.
